# Flocked nasal swab versus nasopharyngeal aspirate for detection of respiratory tract viruses in immunocompromised adults: a matched comparative study

**DOI:** 10.1186/1471-2334-10-340

**Published:** 2010-11-26

**Authors:** Lars Öhrmalm, Michelle Wong, Maria Rotzén-Östlund, Oscar Norbeck, Kristina Broliden, Thomas Tolfvenstam

**Affiliations:** 1 Department of Medicine, Solna, Infectious Disease Unit, Center for Molecular Medicine, Karolinska Institutet, Karolinska University Hospital, SE-171 76 Stockholm, Sweden; 2 Department of Clinical Microbiology, Karolinska University Hospital and Department of Microbiology, Tumor and Cell biology, Karolinska Institutet, Stockholm, Sweden

## Abstract

**Background:**

Several studies have compared nasal swabs to the more invasive nasopharyngeal aspirate (NPA) for detection of respiratory viruses. Mostly, the comparisons have been performed on immunocompetent children with upper respiratory tract symptoms. The results range from a relatively poor sensitivity for the swabs to an even higher sensitivity than for the NPA. We aimed to investigate the sensitivity of a flocked nasal swab (fNS) on immunocompromised adults with febrile neutropenia.

**Methods:**

During 16 months, adults with a hematological disorder presenting with febrile neutropenia were enrolled in the study. Paired samples of the fNS and NPA were collected in the outer part of the nasal cavity and the nasopharynx, respectively. The samples were analyzed regarding a panel of 15 respiratory viruses by means of quantitative polymerase chain reaction. Furthermore, as an indirect measure of cell yield by either method, the copy number of the human beta actin gene was also determined. Cohen's kappa was calculated as a measure of agreement of the results obtained from either method. Wilcoxon signed-rank test was used for comparison of cell yield.

**Results:**

A total of 98 paired samples from a total of 89 patients were collected. Twenty of the pairs had virus detected in at least one of the specimens; 11 in both, 7 in NPA only, and 2 in fNS only. For the fNS, the overall sensitivity for any virus and for rhinovirus only was 65% and 78%, respectively. NPA was significantly superior to the fNS in collecting epithelial cells.

**Conclusion:**

We found the overall sensitivity of 65% to be too low to replace NPA with this sampling technique in this patient category.

## Background

A number of studies have compared different sampling techniques for detection of viruses in the upper respiratory tract in immunocompetent children [1-14]. The advantages of using a swab in the nares compared to nasopharyngeal aspirate (NPA) are for the patient less discomfort and more rapid sampling procedure. For the medical personnel there is a time gain. Finally, the swab goes with a lower cost than does the NPA. Respiratory viruses are common findings in children [15] and adults with hematological malignancies and have been recognized as a potential cause of pneumonia and death [16]. Thus, the sampling frequency for detection of respiratory viruses in this patient category is expected to increase. However, most of the studies comparing sampling techniques are performed on children and, as to our knowledge; to date no study has been performed on immunocompromised individuals which could have a reduced local immunological and inflammatory response, making a direct clinical application of to date achieved results and conclusions impossible. As the swab has been suggested to be comparable with NPA [1,4,9], the primary objective of this study was to determine the sensitivity of detecting respiratory viruses in immunocompromised adults using a flocked nasal swab (fNS) in the outer part of the nose cavity compared to NPA.

## Methods

### Participants

Between January 2008 and May 2009 adults with any hematological disorder presenting at the Karolinska University Hospital, Stockholm, for febrile neutropenia (auricular temperature >38.0°C twice or ≥38.5°C at one occasion, and an absolute neutrophil count ≤500 cells/mm^3^) were asked to participate in this study. The patients were allowed to participate more than once provided that an afebrile period of at least three weeks separated the episodes of febrile neutropenia. At admission, the patients received empirically administrated broad-spectrum antibiotics; ceftazidime or piperacillin-tazobactam.

### Collection and storage of material

The collection was made within 72 hours from onset of fever. The fNS with a nylon fiber tip (COPAN, art. no. CP552C) was inserted at least 20 mm and rotated inside each nostril. Then, the NPA was obtained by insertion of a sterile catheter (no. 8, Mediplast, Sweden) into the posterior nasopharynx and pulled back while applying gentle suction. Finally, 2-3 mL of sodium chloride was sucked into the trap. Both specimens were obtained without instillation of any solution into the nostrils. The specimens were stored without any medium in room temperature and transported to the laboratory within six hours. The NPA was stored in its collection tube in minus 80°C. The fiber tip of the fNS was put in 500 μL RPMI 1640 (Sigma-Aldrich, St. Louis, MO) and shaken for 30 minutes. The suspension was stored in minus 80°C.

### Detection methods

A total of 400 μL of each sample was extracted and then analyzed regarding presence of nucleic acids from adenovirus, bocavirus, coronavirus, enterovirus, influenzavirus A+B, metapneumovirus, parainfluenzavirus 1-3, rhinovirus, and RS-virus. The extraction method and the quantitative polymerase chain reaction (qPCR) assay are previously described [10]. A TaqMan qPCR assay was developed based on amplification of the human beta actin gene (ACTB) (forward primer 5'-GCGCGGCTACAGCTTCA-3' [50 nM], reverse primer 5'-GCGCGGCTACAGCTTCA-3' [900 nM], probe 6FAM-CACCACGGCCGAGC-MGB [150 nM]). The assay was performed on an ABI 7500 Real-Time PCR System (Applied Biosystems). The PCR assay was carried out in a total 50-μL reaction mixture containing 25 μL of TaqMan Universal PCR Master Mix (Applied Biosystems) and 5 μL of template, leaving 20 μL to the primers and probe. The PCR program included 1 cycle at 50°C for 2 min, 1 cycle at 95°C for 10 min, followed by 40 cycles consisting of 15 sec at 95°C and 60 sec at 60°C. The sensitivity of the assay was 3 copies per reaction, as determined by repeated testing of in-house cloned plasmids.

### Ethics

After giving written informed consent the patients were enrolled. The study was approved by The Regional Ethical Review Board in Stockholm that handles applications for research at Karolinska University Hospital.

### Statistical methods

Positivity by either method was used as gold standard for presence of viruses. Cohen's kappa was calculated as a measure of agreement of the results obtained from either method [17]. Wilcoxon signed-rank test and Pearson's correlation coefficient were used when appropriate. Age is presented as means ± SD, whereas neutrophil count is presented as median followed by range. A p-value < 0.05 was considered significant. InStat 3.05 and Prism 5.00 for Windows were used.

## Results

A total of 98 episodes of febrile neutropenia occurring in 89 patients were included in the study. Based on episodes, the mean age was 55 ± 15 years (43 females, 57 ± 13 years; 55 males, 53 ± 16 years) and the median neutrophil count <100 cells/mm^3 ^(<100-500). As underlying hematological disorder, acute leukemia and myelodysplastic syndrome predominated (n = 42, 43%) followed by non-Hodgking lymphoma (n = 33, 34%) and myeloma (n = 13, 13%). The remaining diagnoses were chronic lymphocytic leukemia (n = 6, 6%) and Hodgking's lymphoma (n = 4, 4%). In total, 49 (50%) of the patients were on antiviral prophylaxis with aciclovir. The patients were sampled within one day from admittance (n = 54, 55%), but due to practical obstacles, in 32 (33%) and 9 (9%) of the cases, the samples were collected on the second and third day after admittance, respectively. In total, 98 paired samples collected with fNS and NPA respectively, were obtained. A total of 20 of the pairs had virus detected in one or both of the specimens; 7 exclusively in NPA and 2 exclusively in fNS (Table 1). The sensitivity for the fNS in detection of any virus was 65% (95% CI = 41-85) and for rhinovirus 78% (95% CI = 40-97). The positivity rates for any virus for the NPA and fNS were 18.4% (18 of 98) and 13.3% (13 of 98), respectively, showing a "substantial" agreement (for rhinovirus an "almost perfect" agreement) according to Landis and Koch's scale [17].

**Table 1:** Detection of viruses in NPA and fNS specimens compared with total viral findings by either method

Virus	**No. of samples in which virus was detected by**:			**Total no**.	**Sensitivity**^**a **^**for fNS (%)**	**Mean difference**^**b **^**in Ct values (range)**	**Agreement**^**c **^**between NPA and fNS (95% CI **^**d**^**)**
					
	Both methods	NPA only	fNS only				
Rhinovirus	7	2	0	9	78	3.2 (-2.0-6.5)	0.86 (0.68-1.05)
RSV A	1	1	1	3		5.8	
RSV B	2	0	0	2		2.2 (5.6-9.9)	
Influenza A	0	1	0	1		N/A	
Influenza B	1	1	0	2		15.5	
Bocavirus	0	1	0	1		N/A	
Enterovirus	0	1	0	1		N/A	
Metapneumovirus	0	0	1	1		N/A	
Any virus	11	7	2	20	65	5.1 (-2.0-15.5)	0.66 (0.44-0.87)

**NOTE**. NPA, nasopharyngeal aspirate; fNS, flocked nasal swab; Ct, cycle of threshold; CI, confidence interval.

^a ^Percentage of positive samples from fNS in relation to the total number of pairs with positive results from any of specimens.

^b ^The Ct value for the flocked nasal swab minus the corresponding Ct value for NPA.

^c ^Cohen's kappa: >0.8, almost perfect agreement; 0.6-0.8, substantial agreement; 0.4-0.6, moderate agreement; <0.4, poor agreement.

^d ^CI, confidence interval.

The amount of cells yielded by NPA approximated by ACTB copy numbers was significantly larger than by fNS with a mean difference of 4.2 × 10^5 ^cells/mL (*p *= .001). In pairs with virus detected in both methods, the Ct values were lower (thus viral load higher) for NPA than for fNS. We plotted the difference in cell count between fNS and NPA against the difference in Ct values between the methods for the positive pairs. Although not statistically significant, the correlation indicate a possible association between a high cell yield and low Ct value (r = -0.46, *p *= .15).

## Discussion

We compared the efficacy to collect specimen for viral detection between fNS and NPA. According to Landis and Koch's [17] scale there was a substantial agreement between the methods, to NPA's favor, and NPA was significantly more efficient collecting epithelial material approximated by quantification of ACTB. Even if removal of the cellular component of NPA does not interfere with quantification of some respiratory viruses [18], a higher cell count might indicate a higher yield of specimen. Even though not statistically significant, the Ct value negatively correlated to the number of cells collected, and thus, the finding of NPA being superior to the fNS in collecting cells invite us to speculate that the fNS sometimes may collect inadequate amount of specimen in order to reach the PCR method's detection limit. Furthermore, several reports have suggested that sensitivity increases when the nasopharynx is sampled instead of the nasal cavity [2,5,11,14].

Thus, the difference in sensitivity for the fNS and NPA could have several reasons; biological and technical. One obvious biological reason is the fact that two different anatomical sites are investigated; one technical explanation could be that suction is superior scratching for collecting adequate amount material. The production of mucus that transports the viral nucleic acids in an anterior direction to the fNS's sampling site may be an important parameter. In this patient category, the lack of a competent immune response could reduce the mucus-production. Taking the above into account, the depth of the sampling with the fNS probably plays an important role. However, a deeper penetration could cause even more discomfort than does the NPA.

In contrast to our findings, several groups have reached good sensitivity using the fNS in the outer part of the nose and concluded it comparable to NPA [1,4,9]. Sung et al who used an insertion depth of only 1-1.5 cm summarized the fNS an even better option when using PCR.

This study has several limitations. The time interval between fever onset and sampling varied between the patients, and they did not necessarily have respiratory tract symptoms. Furthermore, the patients suffered from different underlying diseases. However, this reflects the clinical reality and as both sampling methods were used at the same time point in the same patient, the comparison is still valid. The foremost important limitation is the low number of positive samples on which the calculation of sensitivity is based. However, the upper limit in the 95% confidence interval for the overall sensitivity was 85% which is still a questionable value for replacing an established method.

In summary, the NPA was superior to the fNS in collecting epithelial cells. Furthermore, we also found lower sensitivity for the fNS than for NPA in detecting respiratory viruses in these immunocompromised adults. For detection of rhinovirus it was slightly better, but still not appropriate, according to our judgment, for clinical use in this patient category. With an insertion depth comparable to what other groups have used we found lower sensitivity for the fNS than previous investigators. Less mucus production and consequently less anterior transport of viral nucleic acid in immunocompromised individuals may explain this finding.

## Conclusions

On the basis of our findings, fNS sampling of the nares for viral detection in immunocompromised individuals cannot be recommended until further large studies have revealed more promising results.

## Competing interests

The authors declare that they have no competing interests.

## Authors' contributions

All authors have been active in the interpretation of data, the writing of the manuscript and the process of its internal revision as well as the final approval for submission. Furthermore, LÖ has contributed to the study regarding overall design, inclusion of a portion of the patients, acquisition of clinical data, and analysis of data. MW has contributed with laboratory design, compilation, and analysis of data. MRÖ contributed to the study regarding laboratory design, performance of the laboratory work and interpretation of raw data. ON has contributed to the overall design, inclusion of a portion of the patients, and analysis (including statistics) of data. KB was involved in the overall design, and analysis of data. TT contributed to the study regarding initiation and overall design, inclusion of a portion of the patients, acquisition of clinical data and analysis of data.

## Pre-publication history

The pre-publication history for this paper can be accessed here:

http://www.biomedcentral.com/1471-2334/10/340/prepub
